# Pregnancy in Complement-Mediated Thrombotic Microangiopathy: Maternal and Neonatal Outcomes

**DOI:** 10.1016/j.xkme.2023.100669

**Published:** 2023-05-16

**Authors:** Natalja Haninger-Vacariu, Andreas Gleiss, Martina Gaggl, Christof Aigner, Renate Kain, Zoltán Prohászka, Ágnes Szilágyi, Dorottya Csuka, Georg A. Böhmig, Raute Sunder-Plassmann, Gere Sunder-Plassmann, Alice Schmidt

**Affiliations:** 1 Division of Nephrology and Dialysis, Department of Medicine III, Medical University of Vienna, Vienna, Austria; 2 Center for Medical Science, Institute of Clinical Biometrics, Medical University of Vienna, Vienna, Austria; 3 Department of Pathology, Medical University of Vienna, Vienna, Austria; 4 Genetics Laboratory, Department of Laboratory Medicine, Medical University of Vienna, Vienna, Austria; 5 Research Laboratory, Department of Internal Medicine and Hematology, and MTA-SE Research Group of Immunology and Hematology, Hungarian Academy of Sciences and Semmelweis University, Budapest, Hungary

**Keywords:** Thrombotic microangiopathy, aHUS, cTMA, complement system, pregnancy, delivery, neonatal outcomes, pregnancy counseling, live birth, miscarriage, abortion

## Abstract

**Rationale & Objective:**

Pregnancy, delivery, and neonatal outcomes in women with complement-mediated thrombotic microangiopathy (cTMA) have not been well described. A better understanding of these outcomes is necessary to provide women with competent pregnancy counseling.

**Study Design:**

Cohort study.

**Setting and Participants:**

Women with a history of cTMA and pregnancies enrolled into the Vienna thrombotic microangiopathy cohort.

**Exposure:**

New onset or relapses of cTMA.

**Outcomes:**

Pregnancy, delivery, and neonatal outcomes of pregnancies in women (a) before cTMA manifestation, (b) complicated by pregnancy-associated cTMA (P-cTMA), and (c) after first manifestation of cTMA or P-cTMA.

**Analytical Approach:**

Mixed models were used to adjust the comparison of pregnancy, delivery, and neonatal outcomes between conditions (before, with, and after cTMA) for repeated pregnancies using the mother’s ID as random factor. In addition, the fixed factors, mother’s age and neonate’s sex, were used for adjustment. For (sex-adjusted and age-adjusted) centile outcomes, only the mother’s age was used. Adjusted odds ratios were derived from a generalized linear mixed model with live birth as the outcome. Least squares means and pairwise differences between them were derived from the linear mixed models for the remaining outcomes.

**Results:**

28 women reported 74 pregnancies. Despite higher rates of fetal loss before the diagnosis of P-cTMA and preterm births with P-cTMA, most of the women were able to conceive successfully. Neonatal development in all 3 conditions of pregnancies was excellent. Pregnancy and neonatal outcomes were better in women with a pregnancy after the diagnosis of cTMA.

**Limitations:**

Although our data set comprises a considerable number of 74 pregnancies, the effective sample size is lower because only 28 mothers with multiple pregnancies were observed. The statistical power for detecting clinically relevant effects was probably low. A recall bias for miscarriages cannot be ruled out.

**Conclusions:**

Prepregnancy counseling of women with a history of cTMA can be supportive of their desire to become pregnant.

## Graphical abstract

**Figure:**
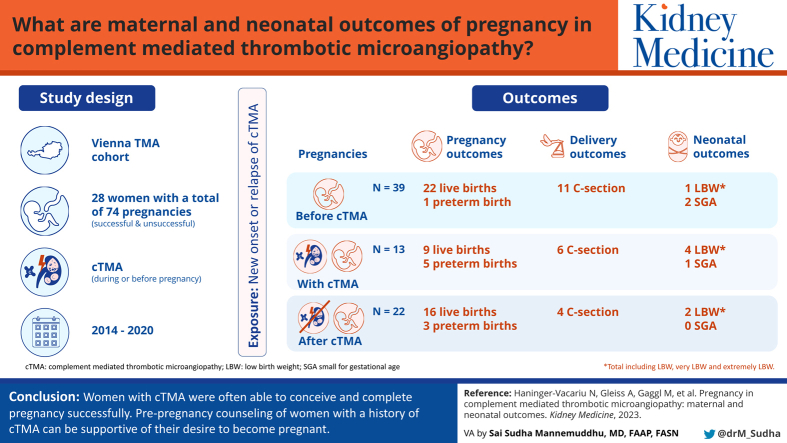

**Plain Language Summary**

A complement-mediated thrombotic microangiopathy (cTMA) related to pregnancy is a life-threatening condition for the mother and the fetus. We studied pregnancy, delivery, and neonatal outcomes of 28 women with a history of cTMA and 74 pregnancies. Despite higher rates of fetal loss before the diagnosis of pregnancy-associated cTMA and preterm births with pregnancy-associated cTMA, most of the women were able to conceive successfully, and neonatal development in all 3 conditions of pregnancies (before, with, and after cTMA) was excellent. Pregnancy and neonatal outcomes were better in women with a pregnancy after diagnosis of cTMA. Thus, women with a history of cTMA can be encouraged to become pregnant.

Complement-mediated thrombotic microangiopathy (cTMA) is a severe complication of pregnancy or the postpartum period.^1^, ^2^, ^3^ Although anticomplement therapy may improve pregnancy and delivery outcomes among such patients, extensive pregnancy counseling is recommended for women with a history of cTMA. Advanced chronic kidney disease, or a history of kidney transplantation including maintenance immunosuppressive therapy, renders decision-making in such cases even more complex.^4^^,^^5^

Sound data on pregnancy, delivery, and neonatal outcomes are needed so that physicians can perform evidence-based prepregnancy counseling in patients with cTMA and help them make informed decisions. Information on pregnancies in women with an established diagnosis of cTMA when compared with that in those complicated by cTMA or with pregnancy outcomes in those before the clinical manifestation of cTMA may be of particular importance. Only a few studies have addressed pregnancy outcomes in patients with an established diagnosis of cTMA, and these studies do not provide sufficient details on delivery and neonatal outcomes.^1^^,^^3^^,^^6^, ^7^, ^8^, ^9^, ^10^, ^11^, ^12^ Of importance, an analysis of pregnancy outcomes before manifestation of cTMA may provide information regarding the rates of miscarriages or stillbirths in patients with a disturbance in the complement system.

To fill this void, we used the database of the Vienna TMA cohort to identify women with cTMA and a history of pregnancy. Then we described and compared pregnancy, delivery, and neonatal outcomes of pregnancies (a) before first cTMA manifestation, (b) with first cTMA triggered by pregnancy (P-cTMA), and (c) after first manifestation of cTMA.

## Methods

### Study Design

This is a cohort study of women with an established diagnosis of cTMA and/or P-cTMA and a history of successful or unsuccessful pregnancies. We described and compared pregnancy, delivery, and neonatal outcomes of pregnancies of the following 3 conditions: (a) before cTMA manifestation, in which pregnancy preceded and was unrelated to the first cTMA manifestations, (b) complicated by P-cTMA, and (c) after first manifestation of cTMA in women with a history of cTMA.

### Setting and Participants

We used the Vienna TMA cohort database to identify women with a history of cTMA who also reported a pregnancy history. The Vienna TMA cohort was established in 2014 at the Division of Nephrology and Dialysis, Department of Medicine III, Medical University of Vienna, Austria, and includes, among other types of TMA, prevalent and incident patients with the diagnosis of cTMA.^3^, ^4^, ^5^^,^^13^, ^14^, ^15^, ^16^, ^17^ For this analysis, we included all women enrolled in the Vienna TMA cohort who reported pregnancies until December 2020. Some of these pregnancies were reported in previous studies.^3^, ^4^, ^5^ The institutional review board (IRB) at the Medical University of Vienna approved the study (unique IRB identifier: 1265/2014). All patients enrolled prospectively in the study gave written informed consent. Investigations were in accordance with the Declaration of Helsinki.

### Pregnancy Outcomes

A pregnancy may result in a live birth, ectopic pregnancy, miscarriage (spontaneous abortion before the 20th week of pregnancy), stillbirth (loss of a fetus after 20 weeks of pregnancy), or an induced abortion. Preterm delivery refers to birth of a live fetus before 37 weeks (extremely preterm: <28 weeks; very preterm: 28-32 weeks; moderate preterm: 32-33 weeks; and late preterm: 34-36 weeks). Infants born in the 37th and 38th weeks of pregnancy are early term, and at 39th or 40th weeks of pregnancy, they are considered full-term. Infants born at 41 weeks through 41 weeks and 6 days are considered late-term and at 42 weeks and beyond as post-term.

### Delivery Outcomes

Among the delivery outcomes, we ascertained spontaneous vaginal delivery; cesarean section; gestational age; birth weight [low birth weight: 2,499 g or less (regardless of the gestational age), very low birth weight: 1,000-1,500 g, and extremely low birth weight: <1,000 g] and birth weight categories (small for gestational age, appropriate for gestational age, and large for gestational age); birth length; head circumference; Apgar scores; admission to a neonatal intensive care unit; and the presence of malformations.

### Neonatal Outcomes

Neonatal outcomes included weight, length, head circumference, developmental delay, and neonatal death within 28 days after delivery. We ascertained weight gain velocity (grams per week), length gain velocity (centimeters per week), and head growth (centimeters per week) from the day of delivery to the day of the first follow-up, approximately week 4 to 7 postpartum.

### Data Sources

Disease-specific data, such as results of genetic testing, a gynecologic history, pregnancy-related outcomes of children and mothers, and neonatal outcomes, were obtained by personal interviews, through a chart review, and from the Austrian Mother-Child Health Passport, which was introduced in 1974, tracking pregnancy-related health information of the mother and child from the beginning of pregnancy until the child’s fifth year.^18^ Participation in this program, which is free of charge even for people without health insurance, is supported by providing financial incentives to the parents. For the assessments at birth of preterm and term infants, we used charts provided by Fenron et al^19^ and Voigt et al,^20^ respectively. Neonatal development was assessed using the World Health Organization growth charts.^21^ On these charts, we defined the normal range as between −2 standard deviation (SD) and +2 SD, represented by *z* scores between −2.0 and +2.0, which correspond by and large to the 2nd and 98th percentiles, respectively. For the calculation of centiles and *z* scores, we used online calculators provided for free by Daniel Gräfe, MD (Leipzig, Germany), accessible on www.pedz.de.

### Statistical Methods

Categorical variables were described as counts and percentages and continuous variables as median and quartiles [interquartile range (IQR)] or range (minimum to maximum). Mixed models were used to adjust the comparison of pregnancy, delivery, and neonatal outcomes between 3 conditions (before, with, and after cTMA) for repeated pregnancies using the mother’s ID as random factor. In addition, fixed factors such as mother’s age and neonate’s sex were used for adjustment. For (sex-adjusted and age-adjusted) centile outcomes, only mother’s age was used. Adjusted odds ratios (aOR) [with 95% confidence intervals (CI)] were derived from a generalized linear mixed model for live birth as the outcome. Least squares means (with 95% CI) and pairwise differences (with 95% Dunnett-corrected CI) between them were derived from linear mixed models for the remaining outcomes. All models were fit to the available data. Conditional model residuals and influence statistics were inspected to identify outliers and potentially influential observations. If necessary, the results of the sensitivity analyses were reported after removing such observations.

*P* values of <0.05 were considered statistically significant. *P* values and CIs for adjusted pairwise group comparisons were corrected using the Dunnett method within each outcome model but were not corrected for testing multiple outcomes, therefore, needing to be interpreted accordingly. All statistical analyses were performed using SAS 9.4 (SAS Institute, 2016).

## Results

### Participants

**Figure 1:**
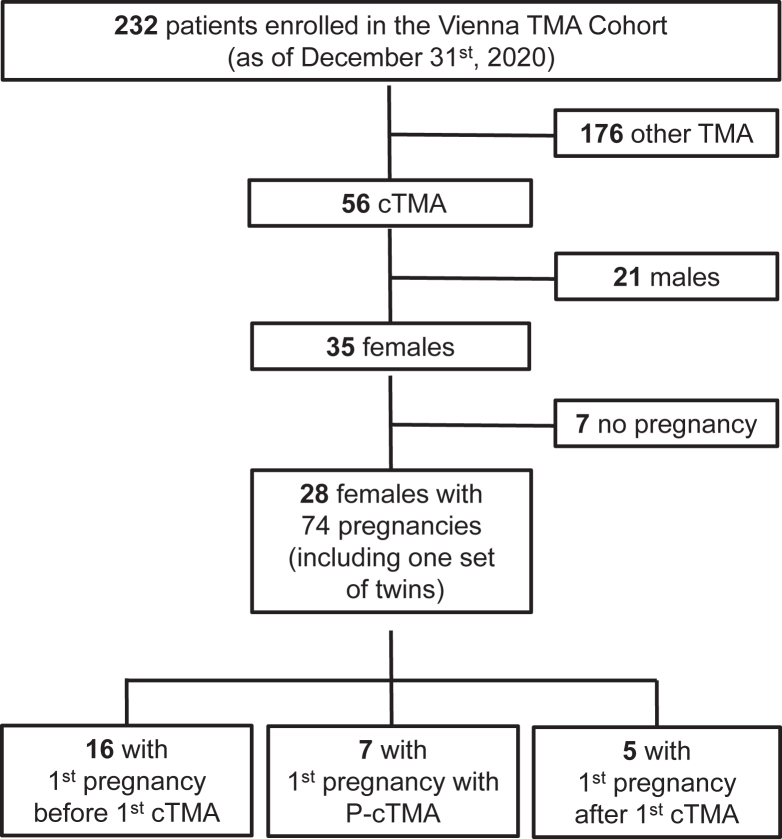
Pregnancies of patients enrolled in the Vienna TMA cohort.

As of December 2020, 28 women diagnosed with cTMA with a history of 74 pregnancies were identified among 232 enrollees of the Vienna TMA cohort database (Fig 1). The median number of pregnancies per woman was 2 (IQR, 1.5-3; range, 1-9). The demographic and clinical data of these 28 women are summarized in Table S1, and the genetic background of cTMA is given in Table S2, including a discussion on the classification of genetic variants. Furthermore, 9 of these women experienced pregnancy with 2 of the 3 conditions (before cTMA manifestation, complicated by cTMA, and after the first manifestation of cTMA), whereas 2 women presented with pregnancies under all 3 conditions.

### Pregnancy Outcomes

**Figure 2:**
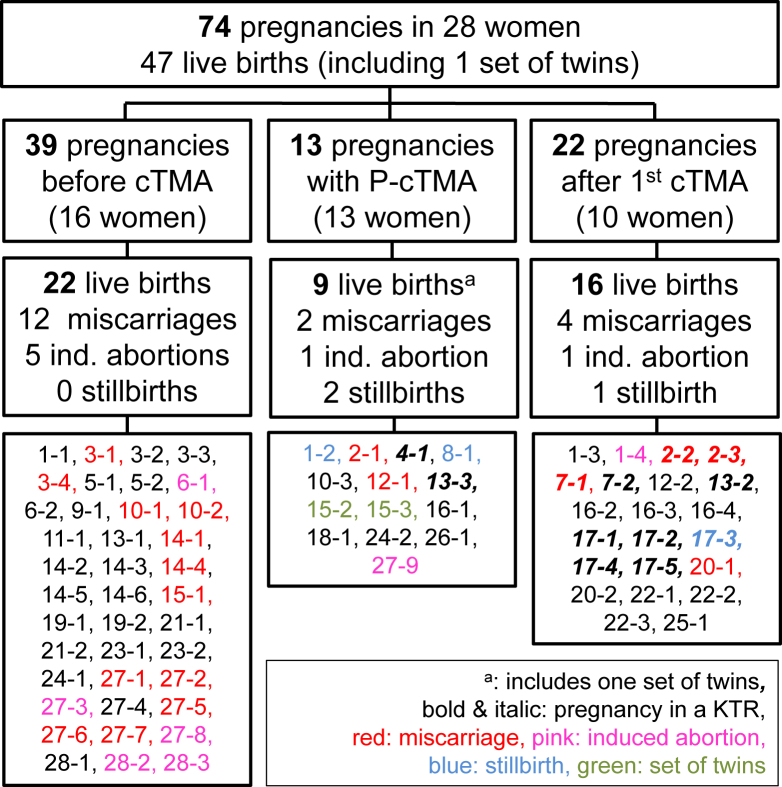
Outcomes for 74 pregnancies of 28 women before cTMA manifestation, of pregnancy-associated cTMA, and of pregnancies after manifestation of cTMA. The first numbers in the 3 boxes at the bottom of the flow chart refer to the patient IDs of 28 women with cTMA and a history of pregnancy.

Details of all 74 pregnancy outcomes are shown in Fig 2. Twelve pregnancies were complicated by first P-cTMA manifestation, 1 pregnancy induced recurrence of cTMA, and 39 pregnancies happened before the diagnosis of cTMA was established. Twenty-two further pregnancies occurred after the manifestation of cTMA and did not trigger a cTMA relapse.

Twenty-six women gave birth to 1-4 children (median, 1.5; IQR, 1-2), and 2 women reported pregnancies without live birth. Three women presented with successful pregnancies with more than 1 condition, and 1 woman presented with 3 successful pregnancies under all 3 conditions. Of the 74 pregnancies, the number of live births recorded was 47 (64%); 22 of the 39 (56%) was before manifestation of cTMA, 9 (including 1 set of twins) of the 13 (64%) with manifestation of cTMA, and 16 of the 22 (73%) after cTMA manifestation or relapse. Known miscarriages or stillbirths were more frequent among pregnancies before cTMA onset (31%) and pregnancy with cTMA (31%) when compared with those among pregnancies after the diagnosis of cTMA (23%). The difference between the 3 conditions regarding the odds of a live birth and adjusted for repeated pregnancies and mother’s age was not statistically significant (*P* = 0.75). The aORs for live births were 37% higher for pregnancies with cTMA and 76% higher for pregnancies after cTMA when compared with pregnancies before cTMA. The aORs for live births were 1.37 (95% CI, 0.29-6.4) for pregnancies with cTMA and 1.76(95% CI, 0.38-8.09) for pregnancies after cTMA, when compared with those for pregnancies before cTMA.

Among 13 women with a history of P-cTMA in our study, 6 reported 14 pregnancies before P-cTMA, with 57% resulting in miscarriage, and 4 women reported 8 pregnancies after P-cTMA, with 25% resulting in miscarriage. By contrast, of the 16 women with a history of cTMA not related to pregnancy, 11 women reported 26 pregnancies before cTMA, with 15% resulting in miscarriage. Six women recorded 14 pregnancies after cTMA, with 14% resulting in miscarriage.

We were not able to identify the reasons for miscarriages. However, 7 abortions were induced: 5 because of unintentional pregnancy, 1 because of malformations, and 1 related to infection. Stillbirth in 3 cases was related to hemolysis, elevated liver enzymes and low platelets syndrome, to intrauterine death owing to pre-eclampsia and infection.

Maternal risk factors for difficult pregnancy and delivery outcomes are indicated in Table S3. Hypertension, proteinuria, and kidney transplantation, but not smoking, were more frequent in pregnancies after cTMA. A history of in vitro fertilization was present in 3 pregnancies with cTMA. Individual data of all pregnancies are summarized in Table S4.

### Delivery Outcomes

Delivery outcomes of 47 live births and the 3 cTMA conditions are given in Table 1. The characteristics of 46 live births according to infant sex (sex of 1 neonate unknown) are given in Table S5, and individual data of all neonates, such as z scores, are given in Table S4.

**Table 1:** Delivery outcomes of 47 live births (raw data)

Pregnancy condition^a^				
	All	Before cTMA	With cTMA	After cTMA
Women, n	28	16	13	10
Pregnancies, n	74	39	13^a^	22
Women with live births, n	26	14	8	9
Live births, n	47^b^	22	9^b^	16
Female, sex, n	19 (40)	7 (32)	6 (67)	6 (38)
Missing	1 (2)	1 (5)		
Gestational age (wk+d)	39 (37-40)	40 (39-41)	35 (32-39)	38 (37-39)
Missing	3 (6)	3 (14)		
Term vs preterm				
Full term	35 (75)	18 (82)	4 (44)	13 (81)
Late preterm	5 (11)	1 (5)	1 (11)	3 (19)
Moderate preterm	1 (2)	0	1 (11)	0
Very preterm	2 (4)	0	2 (22)	0
Extremely preterm	1 (2)	0	1 (11)	0
Missing	3 (6)	3 (14)		
Caesarean section	21 (45)	11 (50)	6 (67)	4 (25)
Induction of labor	9 (35)	3 (27)	0	6 (50)
Missing	2 (4)	2 (9)		
Weight at birth				
Grams	3,200 (3,010-3,560)	3,493 (3,200-3,760)	2,090 (1,280-3,300)	3,130 (3,050-3,375)
Centile	45 (20-59)	45 (17-74)	30 (17-49)	48 (27-55)
Missing	5 (11)	4 (18)	1 (11)	
Birth weight categories				
Normal	35 (75)	17 (77)	4 (44)	14 (88)
Low	3 (6)	1 (5)	0	2 (13)
Very low	3 (6)	0	3 (33)	0
Extremely low	1 (2)	0	1 (11)	0
Missing	5 (11)	4 (18)	1 (11)	
Size for gestational age				
Small	3 (6)	2 (9)	1 (11)	0
Appropriate	36 (77)	16 (73)	6 (67)	14 (88)
Large	3 (6)	0	1 (11)	2 (13)
Missing	5 (11)	4 (18)	1 (11)	
Length at birth				
Centimeter	50 (49-52)	50 (49-52)	44 (38-51)	51 (50-52)
Centile	37 (17-61)	25 (10-55)	23 (16-39)	52 (37-69)
Missing	7 (15)	6 (27)	1 (11)	
Head circumference				
Centimeter	34 (33-35)	34 (33-35)	31 (29-35)	34 (33-35)
Centile	39 (17-54)	33 (16-51)	40 (32-59)	39 (21-52)
Missing	9 (19)	8 (36)	1 (11)	
Apgar score				
1 minute	9 (8-9)	9 (9-9)	8 (8-9)	9 (8-9)
Missing	10 (21)	8 (36)	2 (22)	
5 min	10 (10-10)	10 (10-10)	9 (9-10)	10 (10-10)
Missing	10 (21)	8 (36)	2 (22)	
10 min	10 (10-10)	10 (10-10)	10 (9-10)	10 (10-10)
Missing	12 (26)	11 (50)	1 (11)	
Admission to NICU	7 (15)	0	6 (67)	1 (6)
Missing	2 (4)	2 (9)		
Malformations	7 (15)	3 (14)	1 (11)	3 (19)
Missing	5 (11)	4 (18)	1 (11)	

*Note:* Data are given as n (%) or median (p25, p75).

Abbreviations and definitions: cTMA, complement mediated thrombotic microangiopathy; NICU, neonatal intensive care unit.

a The three pregnancy conditions comprise women (1) before cTMA manifestation, (2) complicated by pregnancy associated cTMA (P-cTMA), and (3) after first manifestation of cTMA or P-cTMA.

b Including one set of twins.

The median (IQR) gestational age of children born before cTMA was 40 weeks (39-41 weeks); 35 weeks (32-39 weeks) with cTMA; and 38 weeks (37-39 weeks) after cTMA. After adjustment for repeated pregnancies, mother’s age, and infant sex, the neonates born from P-cTMA pregnancies showed a younger gestational age (Table 2). After adjustment for cTMA condition and mother’s age, the gestational age of female and male neonates was effectively equal (adjusted mean difference: 0.05 weeks; *P* = 0.95).

**Table 2:** Pregnancy, Delivery, and Neonatal Outcomes of 47 Live Births According to Condition (a) before cTMA, (b) with cTMA, and (c) after cTMA

	Before cTMA (a)	With cTMA (b)	Following cTMA (c)	*P*
Gestational age, wk (95% CI)	39.0 (37.3 to 40.7)	36.5 (34.6 to 38.4)	37.2 (35.4 to 38.9)	0.1
Comparison with (a), wk (95% Dunnett CI)	-	−2.5 (−5.2 to 0.2)	−1.8 (−4.3 to 0.7)	
Gestational age, weeks (95% Dunnett CI)^a^	39.2 (37.9 to 40.6)	36.5 (34.7 to 38.3)	37.9 (36.4 to 39.3)	0.045
Comparison with (a), wk (95% CI)	-	−2.8 (−5.3 to −0.3)	−1.4 (−3.5 to 0.7)	
Birth weight, centile (95% CI)	42.4 (26.6 to 58.3)	39.7 (18.6 to 60.9)	38.5 (21.8 to 55.3)	0.93
Comparison with (a), centile (95% Dunnett CI)	-	−2.7 (−32.9 to 27.6)	−3.9 (−29.6 to 21.8)	
Birth weight, centile (95% CI)^b^	43.4 (28.4 to 58.3)	28.0 (6.0 to 50.1)	42.0 (26.0 to 58.0)	0.47
Comparison with (a), centile (95% Dunnett CI)	-	−15.3 (−46.4 to 15.7)	−1.4 (−25.7 to 22.9)	
Length at birth, centile (95% CI)	33.6 (19.9 to 47.3)	31.2 (12.9 to 49.6)	51.2 (37.5 to 65.0)	0.1
Comparison with (a), centile (95% Dunnett CI)	-	−2.4 (−29.0 to 24.2)	17.6 (−4.4 to 39.6)	
Head circumference, centile (95% CI)	35.2 (18.0 to 52.4)	46.3 (24.8 to 67.8)	37.4 (20.8 to 54.0)	0.68
Comparison with (a), centile (95% Dunnett CI)	-	11.1 (−20.5 to 42.7)	2.2 (−24.6 to 29.0)	
Head circumference, centile (95% CI)^b^	36.1 (20.3 to 51.8)	35.0 (13.0 to 56.9)	40.6 (25.4 to 55.8)	0.86
Comparison with (a), centile (95% Dunnett CI)	-	−1.1 (−32.7 to 30.5)	4.5 (−20.1 to 29.2)	
Weight gain velocity, g/d (95% CI)	199 (125 to 274)	204 (120 to 287)	252 (182 to 321)	0.39
Comparison with (a), g/d (95% Dunnett CI)	-	4 (−121 to 130)	52 (−55 to 159)	
Weight gain velocity, g/d (95% CI)^c^	196 (155 to 237)	228 (171 to 284)	219 (179 to 260)	0.55
Comparison with (a), (95% Dunnett CI)	-	32 (−50 to 114)	23 (−43 to 89)	

Abbreviations and definitions: cTMA, complement mediated thrombotic microangiopathy; CI, confidence interval (corrected for two pairwise comparisons within each outcome model using Dunnett’s method).

a Excluding pregnancy 4-1 (extreme preterm) and pregnancy 16-1 (large for gestational age).

b Excluding pregnancy 16-1 (large for gestational age).

c Excluding pregnancy 4-1 (extreme preterm) and 25-1 (rapid growth).

Growth chart centiles for weight, length, and head circumference were as follows: the median centiles (IQR) for weight at birth for the 3 different pregnancy conditions were 45th (17-74) before cTMA; 30th (17-49) with cTMA; and 48th (27-55) after cTMA. The median centiles (IQR) for length at birth were 25th (10-55) before cTMA; 23rd (16-39) with cTMA; and 52nd (37-69) after cTMA. The median centiles (IQR) for head circumference at birth were 33rd (16-51) before cTMA; 40th (32-59) with cTMA; and 39th (21-52) after cTMA. Least square means and corresponding 95% CIs for gestational age, weight, length, and head circumference, after adjustment for repeated pregnancies, mother’s age, neonate’s sex, and the differences between the pregnancy conditions are summarized in Table 2.

We found clinically relevant differences in the delivery outcomes regarding temporality of onset of cTMA (Table 1): there were more girl babies, a lower weight at birth, and more admissions to the neonatal intensive care unit in neonates born from pregnancies complicated by cTMA.

### Neonatal Outcomes

**Figure 3:**
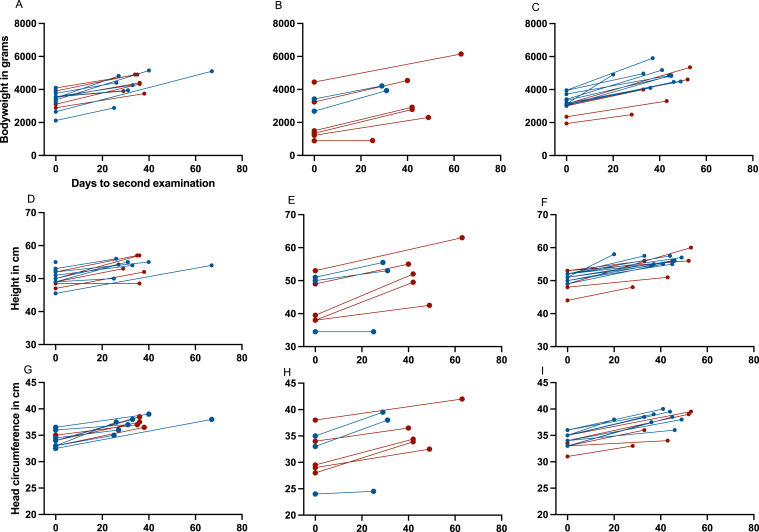
Development of neonatal weight, length, and head circumference according to timing of pregnancies. Days to second examination are shown on the X-axis.

Overall, the neonatal development was worse in infants born from pregnancies complicated by P-cTMA. The *z* scores <−2 for weight, length, and head circumference were more frequent in this condition at birth and at follow-up (Table S4). Details of weight, length, head circumference, and other important variables over time are provided in Table S6a-c for all 3 pregnancy conditions. Weight and length gain velocity and head growth of infants born before, with, or after cTMA (Table S6a-c). Figure 3 shows neonatal development according to sex in all 3 pregnancy conditions and individual data of all neonates are given in Table S4.

The median weight gain velocity (IQR) (in g/d)for the before, with, and after cTMA pregnancy conditions was 175 (158-239), 210 (172-240), and 210 (189-293), respectively. Least square means and corresponding 95% CIs, after adjustment for repeated pregnancies, mother’s age, neonate’s sex (not significant), and the differences between pregnancy conditions (not significant) are summarized in Table 2. Weight gain in female neonates was 39.2 g/d lower when compared with the male neonates (*P* = 0.12, adjusted for cTMA condition and mother’s age).

## Discussion

In this study of pregnancies in women with a history of cTMA, we showed that infants born from a pregnancy complicated by cTMA have a significantly lower gestational age when compared with the neonates born before or after the diagnosis of cTMA in the mother. Moreover, these children presenting with lower weight at birth, are more likely to be female, and are frequently in need of intensive medical care. Of importance, pregnancies after an established diagnosis of cTMA in the mother seem to be more successful when compared with pregnancies before diagnosis of P-cTMA, which more frequently results in miscarriages.

Pregnancy outcomes (ie, live birth or not) have been reported among patients with P-cTMA or cTMA not related to a pregnancy in several studies.^1^^,^^3^^,^^6^, ^7^, ^8^, ^9^, ^10^ In addition, delivery outcomes (gestational age and weight at birth) have been summarized in some studies.^3^^,^^6^, ^7^, ^8^^,^^10^ However, neonatal outcomes were considered only in a few patients in 3 of these studies.^3^^,^^6^^,^^8^

Although other studies focused primarily on maternal and pregnancy outcomes, and genetics and complement therapies in women with P-cTMA, the main objectives of our study included miscarriages and neonatal outcomes of women with pregnancies before manifestation of cTMA, with P-cTMA and, importantly, pregnancies after an established diagnosis of cTMA.

The overall live birth rate in this study was 64%, comparing with other studies.^1^^,^^3^^,^^6^, ^7^, ^8^, ^9^, ^10^ Among 9 women, there was a history of 18 miscarriages and, among 3 women, a history of 3 stillbirths. Among women with a history of P-cTMA in this study, pregnancies resulted in miscarriage in 57% of the cases before P-cTMA and in 25% after P-cTMA. By contrast, in women with a history of cTMA not related to pregnancy, pregnancies resulted in miscarriage in 15% of pregnancies before cTMA and in 14% after cTMA, comparing with the rate of recognized miscarriages in the general population.^22^ Thus, complement dysregulation resulting in P-cTMA is also associated with a higher risk for miscarriage before cTMA manifestation when compared with cTMA not related to pregnancy. This observation seems to be related to an insufficient complement inhibition at the maternal-fetal interface that prevents inappropriate complement activation to protect the fetus during normal pregnancy.^23^ Moreover, all younger patients with cTMA not related to pregnancy who tried to conceive later on showed at least 1 successful pregnancy after cTMA onset.

Regarding delivery outcomes, 75% of all neonates in our study were full-term, 75% showed a birth weight >2,500 g, and the size was appropriate for gestational age in 79%. However, delivery outcomes were different between the 3 pregnancy groups.

Data on neonatal outcomes of successful pregnancies in patients with cTMA are scarce. Huerta et al^6^ described a 500-g neonate at week 24 of gestation who developed well during the following 5 months of hospital stay.^6^ Furthermore, Timmermans et al^8^ reported 2 neonatal deaths at day 3 and at day 4 in 1 pregnancy not complicated by cTMA and in 1 case of P-cTMA.^8^ The main focus of our study was the detailed analysis of neonatal development of children born to mothers with a diagnosis of cTMA. Overall, neonatal development was satisfactory in all 3 pregnancy conditions (Figure 3).

In this study, 16 women reported 39 pregnancies before the diagnosis of cTMA (Figure 2). Five of 16 (31%) presented with a history of 12 miscarriages (of 39 pregnancies, 31%) and 4 women a history of 5 induced abortions (of 39 pregnancies, 13%). In comparison with our study, Meibody et al,^9^ Huerta et al,^6^ and Bruel et al^10^ described miscarriages in 5 of the 14 (36%), 3 of the 8 (38%), and 10 of the 49 (20%) women with a later diagnosis of cTMA.^6^^,^^9^^,^^10^ Thus, in line with Fakhouri et al,^1^ we provide further evidence that complement dysregulation is involved in pregnancy complications such as pre-eclampsia and fetal loss.^1^ In this regard, all 5 women of our study with a history of miscarriage before the diagnosis of cTMA showed variants in complement-associated genes (Fig 2; Table S2, patient ID: 3, 10, 14, 15, and 27). As discussed earlier, the miscarriage rate of women in this pregnancy group, with a later diagnosis of P-cTMA, was >50%. However, delivery outcomes and neonatal outcomes were excellent in this group when compared with those in the group of pregnancies complicated by P-cTMA.

Among the pregnancies complicated by cTMA in this study, 9 of the 13 (69%) resulted in a live birth, comparing well with outcomes from other series in the literature.^1^^,^^3^^,^^6^, ^7^, ^8^^,^^10^ For further comparison, 5 (56%) neonates in our study group were preterm, and 1 (11%) was small for gestational age. Birth weight and length were in the lower centiles range (Table 1), which points to intrauterine developmental delay of a fetus from a cTMA pregnancy, although there are no clinical signs of cTMA during these pregnancies. In this study, 33% of the women underwent in vitro fertilization, and Meibody et al^8^ also reported that in vitro fertilization was used by 2 of the 14 pregnancies in patients with cTMA.^9^ Further research is necessary to determine whether infertility is related to complement disturbance or in vitro fertilization creates a complement or coagulation environment that serves as an additional trigger factor for the development of cTMA.^23^

In comparison with pregnancies before or after a diagnosis of cTMA, neonates born from P-cTMA pregnancies showed a significantly lower gestational age and were more likely to have a lower birth weight, be female, and require postpartum intensive care. Our observation of more preterm female neonates born from P-cTMA pregnancies is in line with findings of a systematic review and meta-analysis of 12.5 million women, which showed female sex to be associated with preterm pre-eclampsia. At term, this association is reversed, and male fetal sex is associated with pre-eclampsia.^24^ However, neonatal development was not affected in the children of our study, although low *z* scores for weight and length were observed in many of them at birth and at follow-up (Fig 3).

There are no sound data available on the success of pregnancies in women with a history of cTMA. In this study, 22 pregnancies occurred in 10 women after an established diagnosis of cTMA, none of which enabled by in vitro fertilization. Sixteen of the 22 pregnancies resulted in live births (73%) from 9 women, and most (90%) of the women conceived successfully. Five of the 22 pregnancies (23%) from 4 women resulted in fetal loss; however, the rate of miscarriage after cTMA diagnosis was lower when compared with that of pregnancies before (31%) or with cTMA (31%). Furthermore, 4 of these women showed a history of P-cTMA, and 8 later pregnancies resulted in 5 live births, 2 miscarriages, and 1 induced abortion. Of note, among 8 (50%) successful pregnancies, eculizumab was used in 2 cases and plasma therapy in 6 cases; however, we observed 1 miscarriage despite eculizumab therapy in 1 woman. In addition, 6 of the successful pregnancies were reported among kidney transplant recipients, a situation that renders pregnancy even more complicated.^5^ Only 3 of the 16 (19%) neonates were preterm, and none were small for the gestational age. Neonatal development was also excellent in these children, with *z* scores for weight and length in the expected range for average development.

The lack of comprehensive delivery and neonatal outcome data in other analyses may be due to the registry nature of several previous studies. In this study, the completeness of data is supported by the Austrian mother-child book that collects pregnancy and neonatal data and by numerous repeated interviews with our patients, which is made possible by the single center nature of our study. However, a recall bias regarding previous miscarriages cannot be ruled out. Furthermore, among patients with an established diagnosis of cTMA, we provide prospectively collected data of all pregnancies from the past decade. Although our data set comprises a considerable number of pregnancies, the effective sample size is lower because only 28 mothers with multiple pregnancies were observed. Our analysis considered this within-mother correlation using a random effect, but statistical power for detecting clinically relevant effects is probably low.

In conclusion, women who were pregnant before being diagnosed with P-cTMA experienced higher rates of fetal loss than women who had a pregnancy after diagnosis. In addition, neonates born to women with P-cTMA were more likely to be preterm. However, most of the prospectively followed up women with a history of P-cTMA and cTMA were able to conceive successfully, and neonatal development in all 3 categories of pregnancies (before, with, and after cTMA) was excellent. Pregnancy and neonatal outcomes were improved in women with a pregnancy after diagnosis. Therefore, prepregnancy counseling of women with a history of cTMA can be supportive of their desire to become pregnant.
