# Supplementary material for: Pregnancy in Complement-Mediated Thrombotic Microangiopathy: Maternal and Neonatal Outcomes
Source: Kidney Med. 2023 May 16;5(7):100669. doi: 10.1016/j.xkme.2023.100669 (PMC10363558; doi:10.1016/j.xkme.2023.100669)
Supplement: Supplementary File (PDF) — Table S1-S6. [file mmc1.pdf]

**Table S1.** Demographic and clinical data of 28 women with a history of cTMA and pregnancy.

| | | Pregnancy condition | | |
|---|---|---|---|---|
| Characteristic | All | Before cTMA | With cTMA | After cTMA |
| Women | 28 | 16 | 13 | 10 |
| Pregnancies | 74[a] | 39 | 13[a] | 22[b] |
| Pregnancies before 2012[c] | 40 (54) | 30 (77) | 5 (38) | 5 (23) |
| Age at delivery | 29 (24, 34) | 27 (20, 32) | 30 (26, 34) | 30 (28, 34) |
| Age at diagnosis of cTMA | 30 (23, 40) | 38 (29, 52) | 28 (24, 34) | 20 (13, 24) |
| Biopsy confirming TMA | 19 (68) | 11 (69) | 8 (62) | 6 (60) |
| Eculizumab use | | | | |
| Continuous before-during-after pregnancy | 2 (7) W/3 (4) P | - | - | 2 (20) W/3 (14) P |
| At pregnancy for postpartum P-cTMA | 4 (14) W/4 (5) P | - | 4 (31) W/4 (31) P | - |
| Not related to pregnancy | 8 (29) W | 8 (50) W | - | - |
| Plasma exchanges or infusions | | | | |
| Continuous before-during-after pregnancy | 4 (7) W/7 (10) P | - | 1 (8) W/1 (8) P (switch to ecu) | 4 (40) W/6 (27) P |
| At pregnancy for postpartum P-cTMA | 7 (25) W/7 (10) P | - | 7 (54) W/7 (54) P (2 x switch to ecu) | - |
| Not related to pregnancy | 7 (25) W (6 x switch to ecu) | 7 (44) W (6 x switch to ecu) | - | - |
| Impaired kidney function before pregnancy | 8 (29) W/16 (22) P | - | 2 (15) KTR/2 (15) P | 7 (70) W/14 (64) P |
| Dialysis at last follow-up | 4 (14) | 1 (6) | 3 (23) | 1 (10) |
| Kidney transplant at last follow-up | 8 (29) | 6 (38) | 2 (15) | 3 (30) |
| Deaths | 4 (14),2 HD, 2 KTR | 2 (13) KTR | 2 (15) HD | 1 (10) HD |
| Age at death, years | 49 (41-55) | 44-55 | 41-54 | 54 |

Data are given as n (%) or median (p25, p75)

[a], includes one set of twins; [b], 3 women received aspirin during 3 pregnancies; [c], before use of eculizumab in our center

cTMA, complement mediated thrombotic microangiopathy; KTR, kidney transplant recipient; HD, hemodialysis

W, women; P, pregnancies; ecu, eculizumab

**Table S2**. Genetic variants, risk haplotypes, and copy number variations of 28 women with cTMA and a history of pregnancy.

| Patient ID | Gene | Variant[a] | MAF %[b] (gnomAD global) | MAF %[c] (1000G global) | Risk haplotype | (Variants) Copy number of *CFHR-1,2,3,5* |
|---|---|---|---|---|---|---|
| 1 | - | - | - | - | *CFH* H3, het; *CD46*ggaac, het | WT |
| 2 | *THBD* | p.E560Q het (LPV) | 0.0018% | 0.02% | *CFH* H3, het | WT |
| 3 | *CFHR5* | p.G228A het (VUS) | 0.0016% | 0 | *CFH* H3, hom | WT |
| 4 | - | - | - | - | *CFH* H3, het; *CD46*ggaac, hom | WT |
| 5 | - | - | - | - | *CD46*ggaac, hom | WT |
| 6 | *C3* | p.D61N het (VUS) | 0.002% | 0 | *CFH* H3, het; *CD46*ggaac, hom | WT |
| 7 | *CFH* | p.C1032* het (PV) | 0 | 0 | *CD46*ggaac, hom; *CFH* H8, het | het del*CFHR1,3* |
| 8 | - | - | - | - | *CFH* H3, hom; *CD46*ggaac, het | WT |
| 9 | - | - | - | - | *CFH* H3, het | WT |
| 10 | *CD46* | p.E142Q het (VUS) | 0.008% | 0 | *CFH* H3, het | het del*CFHR1,3* |
| 11 | - | - | - | - | - | het del*CFHR1,3* |
| 12 | *CFHR5* | c.479_480insA het (VUS) | 0.2% | 0.08% | *CD46*ggaac, het | WT |
| 13 | *CFH* | p.N516K het (VUS) | 0.03% | 0.02% | *CFH* H3 het; *CFH* H8, het *CD46*ggaac, hom | WT |
| 14 | - | - | - | - | *CFH* H3, het; *CD46*ggaac, hom | WT |
| 15 | - | - | - | - | *CFH* H3, het; *CD46*ggaac, hom | WT |
| 16 | *C3*<br>*C3* | p.K104E het (VUS)<br>p.D1457H het (VUS) | 0<br>0.03% | 0<br>0.16% | *CFH* H3, het; *CD46*ggaac, het | WT |
| 17 | *CFI* | p.I416L het (VUS) | 0.03% | 0.09% | *CD46*ggaac, het | het del*CFHR1,3* |
| 18 | *C3* | p.V1296A het (VUS) | 0 | 0 | *CFH* H3, het; *CD46*ggaac, het | het del*CFHR1,3* |
| 19 | - | - | - | - | *CFH* H3, het; *CD46*ggaac, hom | n.a. |
| 20 | - | - | - | - | *CFH* H3, het | WT |
| 21 | - | - | - | - | *CFH* H3, hom; *CD46*ggaac, het | WT |
| 22 | *CFI*<br>*CD46* | p.G342E het (LPV)<br>p.D257Vfs41 het (LPV) | 0.0012%<br>0 | 0<br>0 | *CFH* H3, het; *CD46*ggaac, het | WT |
| 23 | *CD46* | p.E234K het (VUS) | 0.0056% | 0.02% | *CFH* H3, het; *CD46*ggaac, het | n.a. |
| 24 | *CFH* | p.D748Nfsa10 het (PV) | 0 | 0 | *CFH* H3, het; *CD46*ggaac, het | WT |
| 25 | - | - | - | - | *CD46*ggaac het | het del*CFHR1,3* |
| 26 | - | - | - | - | - | het del*CFHR1,3* |
| 27 | - | - | - | - | *CD46*ggaac, het | n.a. |
| 28 | *CFH*<br>*CFH* | p.S1191L het (PV)<br>p.V1197A het (PV) | 0<br>0.0004% | 0<br>0 | *CD46*ggaac, het | elevated signal of *CFHR1* exon 6 due to mutations in *CFH* (resulting from gene conversion between *CFH* and *CFHR1)* |

[a], Variant classification according to the American College of Genetics and Genomics in parenthesis
[b], Minor allele frequency (MAF) of the variant allele in the overall (global) gnomAD population (https://gnomad.broadinstitute.org/)
[c], Minor allele frequency (MAF) of the variant allele in the overall (global) 1000Genomes population (https://www.internationalgenome.org/)

cTMA, complement mediated thrombotic microangiopathy; het, heterozygous; hom, homozygous; PV, pathogenic variant; LPV, likely pathogenic variant; VUS, variant of unknown significance; WT, wild-type; n.a., not analyzed

## Table S2, discussion.

Papers reporting on the genetic background of pregnancy-associated aHUS usually list all the identified rare complement gene variants, including those classified as VUS. In our cohort, there were 5 cases, or 17% of total patients, with variations classified as PV or LPV (patient 2, 7, 22, 24 and 28). There were a further 7 patients with variations classified as VUS in the *CFH*, *CFI*, *CD46* or *C3* genes. Altogether, this is 42% of all patients with a rare complement gene variation, which is comparable to previous reports.

A multicenter study (France, United Kingdom, Italy, PMID: 28596415) aimed to identify presentation, outcomes, and frequency of complement alternative pathway gene variants with an international cohort of 87 patients with p-HUS. Novel or rare variants of complement genes were present in 56% (49/87) of women and this list included VUS variations as well (for example the *CFH* p.N516K identified in our cohort as well, or the *CFI* p.I416L which they assessed as pathogenic).
In a retrospective study of the Spanish P-aHUS Registry (PMID: 28911789), 9 out of 22 patients (41%) were reported to carry a complement abnormality in the aHUS-associated candidate genes, although not all of the listed variants are pathogenic based on the current ACMG guidelines *(*e.g. *C3* p.S1619R is likely benign according to the complement database, www.complement-db.org).
A further study by Rondeau et al. (PMID: 34515154) recorded that pathogenic variants were identified in 20 out of 33 (60.6%) tested aHUS patients who became pregnant. Among them, patients carrying only risk haplotypes/polymorphisms were also listed; there were only 7 patients with (L)PV variations, according to the classification of complement-database.org.
In a recent study by Fakhouri et al. (PMID: 33826112), the proportion of women with pregnancy-associated aHUS carrying pathogenic variant(s) in complement genes and/or anti-CFH antibody was found to be 45.1% (23/51). The detailed list of the identified pathogenic variations was not available for review.

Of note, conflicting interpretations of pathogenicity are available for some of the variations listed as VUS in our paper.

The *CFI* p.I416L variation - classified as pathogenic in PMID: 28596415 - was reported to dramatically decrease the FI level in cell culture supernatants as detected by enzyme-linked immunosorbent assay (ELISA) and western blotting, as well as to reduce the amount of intracellular FI. (PMID: 20016463). It was identified in several aHUS patients, however, based on the study by Osborne et al (PMID: 29500241), the allele frequency in aHUS patients was not found to be higher than the MAF in healthy populations, thus the complement database classified it as of uncertain significance.
The *CD46* p.E234K was reported as an aHUS associated mutation in PMID: 26054645. However, as no other appearance of the corresponding variation was described in the literature and its frequency is 0.0056% in gnomAD and 0.02% in 1000Genomes, we classified it as VUS.
In addition, mutations that are absent from (*C3* p.V1296A, *C3* p.K104E) or have very low frequency in large population studies (*C3* p.D61N, gnomAD: 0.002%, 1000Genome: 0), were classified as of uncertain significance, because we did not find records of them in other complement mediated TMA patients and no data is available on their functional role.

References:
PMID: 28596415, Bruel A, Kavanagh D, Noris M, et al. Hemolytic uremic syndrome in pregnancy and postpartum. Clin J Am Soc Nephrol 2017; 12: 1237–1247.
PMID: 28911789, Huerta A, Arjona E, Portoles J, et al. A retrospective study of pregnancy-associated atypical hemolytic uremic syndrome. Kidney Int 2018; 93: 450–459.
PMID: 34515154, Eric Rondeau et al, Pregnancy in Women with Atypical Hemolytic Uremic Syndrome. Nephron. 2022;146(1):1-10.
PMID: 33826112, Fakhouri et al. Pregnancy-triggered atypical hemolytic uremic syndrome (aHUS): a Global aHUS Registry analysis. J Nephrol. 2021 Oct;34(5):1581-1590.
PMID: 20016463, Bienaime et al. Mutations in components of complement influence the outcome of Factor I-associated atypical hemolytic uremic syndrome. Kidney Int. 2010 Feb;77(4):339-349.
PMID: 29500241,Osborne et al. Statistical validation of rare complement variants provides insights into the molecular basis of atypical hemolytic uremic syndrome and C3 glomerulopathy. J Immunol. 2018 Apr;200(7):2464-2478.

**Table S3.** Maternal risk factors for worse delivery outcomes of 47 live births.

| | | Pregnancy condition | | |
|---|---|---|---|---|
| Characteristic | All | Before cTMA | With cTMA | After cTMA |
| Women with live births, n | 26 | 14 | 8 | 9 |
| Live births, n | 47[a] | 22 | 9[a] | 16 |
| In vitro fertilization | 3 (6) | 0 | 3 (33) | 0 |
| Current smoking | 15 (32) | 11 (50) | 2 (22) | 2 (13) |
| Hypertension [b] | 20 (43) | 2 (9) | 5 (56) | 13 (81) |
| Proteinuria [b] | 19 (40) | 2 (9) | 4 (44) | 13 (81) |
| Impaired kidney function [c] | 14 (30) | 0 | 2 (22) | 12 (75) |
| Pregnancies in KTRs | 8 (17)[d] | 0 | 2 (22)[e] | 6 (38)[f] |

Data are given as n (%)

[a], includes one set of twins; [b], during pregnancy; [c], elevated serum creatinine or proteinuria before pregnancy; [d], five women; [e], two women; [f], three women

cTMA, complement mediated thrombotic microangiopathy; KTR, kidney transplant recipient

**Table S4.** Individual delivery and neonatal outcomes of 74 pregnancies in 28 women with cTMA.

| Mother and pregnancy ID | Pregnancy before, with, after cTMA | Pregnancy outcome | Gestational age (week+days) | Time of delivery | Mode of delivery | Infants sex | Birth weight (g) | Birth weight assessment | Birth weight centile | Birth weight z-score | Birth length (cm) | Birth length centile | Birth length z-score | Birth head circumference (cm) | Birth head circumference centile | Birth head circumference z-score | Apgar score (1 minute) | Apgar score (5 minutes) | Apgar score (10 minutes) | Admission to NICU | Malformations* | Weight at follow-up (g) | Weight at follow-up centile | Weight at follow-up z-score | Length at follow-up (cm) | Length at follow-up centile | Length at follow-up z-score | Head circumference at follow-up (cm) | Head circumference at follow-up centile | Head circumference at follow-up z-score | Breastfeeding | Age at follow-up (days) | Infants age as of December 2020 (years) |
|---|---|---|---|---|---|---|---|---|---|---|---|---|---|---|---|---|---|---|---|---|---|---|---|---|---|---|---|---|---|---|---|---|---|
| 1-1 | Before | L | 35+6 | Late PT | C section | m | 2117 | Low BW | 9,00 | -1,35 | 49 | 79,00 | 0,81 | 33,0 | 62,00 | 0,30 | 7 | 9 | 9 | - | - | 2880 | 16 | -1,00 | 50,0 | 52 | 0,06 | 35,0 | 66 | 0,42 | - | 25,0 | 8,3 |
| 1-2 | With | S | 33+1 | Moderate PT | Vaginal | f | 1860 | Low BW | | | 41 | | | 29,0 | | | | | | | | | | | | | | | | | | | |
| 1-3 | After | L | 35+0 | Late PT | Vaginal | f | 2350 | Low BW | 49,00 | -0,03 | 48 | 85,00 | 1,04 | 33,0 | 85,00 | 1,03 | 8 | 10 | 10 | - | - | 3300 | 42 | -0,21 | 51,0 | 57 | 0,18 | 34,0 | 26 | -0,64 | - | 43,0 | 1,5 |
| 1-4 | After | A (i) | <12 | | | | | | | | | | | | | | | | | | | | | | | | | | | | | | |
| 2-1 | With | M | 18 | | | | | | | | | | | | | | | | | | | | | | | | | | | | | | |
| 2-2 | After | M | 7 | | | | | | | | | | | | | | | | | | | | | | | | | | | | | | |
| 2-3 | After | M | 9+6 | | | | | | | | | | | | | | | | | | | | | | | | | | | | | | |
| 3-1 | Before | M | <12 | | | | | | | | | | | | | | | | | | | | | | | | | | | | | | |
| 3-2 | Before | L | 40 | Full term | Vaginal | m | 2650 | Normal BW | 1 | -2,21 | 46 | 0,99 | -3,04 | 32,5 | 1 | -2,38 | 9 | 10 | na | - | - | 5100 | 15 | -1,04 | 54,0 | 0,99 | -2,61 | 38,0 | 10 | -1,3 | na | 67,0 | 21,0 |
| 3-3 | Before | L | 40 | Full term | Vaginal | f | 3100 | Normal BW | 19 | -0,88 | 49 | 7 | -1,45 | 34,0 | 25 | -0,69 | 9 | 10 | na | - | - | 4330 | 59 | 0,22 | 52,5 | 26 | -0,63 | 38,5 | 95 | 1,64 | na | 36,0 | 18,5 |
| 3-4 | Before | M | <12 | | | | | | | | | | | | | | | | | | | | | | | | | | | | | | |
| 4-1 | With | L | 27+2 | Extremely PT | C section | f | 888 | Extremely low BW | 42,00 | -0,20 | 35 | 47,00 | -0,08 | 24,0 | 39,00 | -0,29 | 5 | 7 | 9 | Yes | - | 900 | 0,99 | -7,14 | 34,5 | 0,99 | -7.86 | 24,5 | 0,99 | -7.92 | - | 25,0 | 19,0 |
| 5-1 | Before | L | 41+0 | Full term | C section | m | 3270 | Normal BW | 15 | -1,04 | 52 | 32 | -0,48 | 36,0 | 53 | 0,08 | na | na | na | - | - | na | na | na | na | na | na | na | na | na | Yes | na | 39,1 |
| 5-2 | Before | L | 40+5 | Full term | Vaginal | f | 3500 | Normal BW | 46 | -0,11 | 50 | 17 | -0,94 | 34,0 | 19 | -0,86 | 9 | 10 | 10 | - | Yes | 4400 | 39 | -0,29 | 57,0 | 84 | 0,98 | 37,5 | 58 | 0,2 | - | 36,0 | 31,2 |
| 6-1 | Before | A (i) | na | | | | | | | | | | | | | | | | | | | | | | | | | | | | | | |
| 6-2 | Before | L | 38+3 | Full term | C section | m | 3365 | Normal BW | 48 | -0,05 | 52 | 59 | 0,24 | 36,5 | 85 | 1,05 | 9 | 10 | 10 | - | Yes | 5150 | 85 | 1,05 | 55,0 | 54 | 0,11 | 39,0 | 93 | 1,45 | Yes | 40,0 | 15,0 |
| 7-1 | After | M | 12 | | | | | | | | | | | | | | | | | | | | | | | | | | | | | | |
| 7-2 | After | L | 37+0 | Full term | Vaginal | m | 3082 | Normal BW | 48 | -0,04 | 50 | 47 | -0,08 | 34,0 | 39 | -0,27 | 8 | 10 | 10 | - | - | 4460 | 68 | 0,47 | 56,0 | 87 | 1,14 | 36,0 | 26 | -0,65 | Yes | 46,0 | 1,9 |
| 8-1 | With | S | 31+1 | Very PT | C section | m | 1210 | Very low BW | | | na | | | na | | | | | | | | | | | | | | | | | | | |
| 9-1 | Before | L | na | na | C section | f | na | na | na | na | na | na | na | na | na | na | na | na | na | - | na | na | na | na | na | na | na | na | na | na | na | na | 29,3 |
| 10-1 | Before | M | 6 | | | | | | | | | | | | | | | | | | | | | | | | | | | | | | |
| 10-2 | Before | M | 7 | | | | | | | | | | | | | | | | | | | | | | | | | | | | | | |
| 10-3 | With | L | 32+0 | Moderate PT | C section | f | 1220 | Very low BW | 11,00 | -1,25 | 38 | 11,00 | -1,23 | 29,0 | 54,00 | 0,11 | 8 | 9 | 9 | Yes | - | 2300 | 1 | -2,25 | 42,5 | 0,99 | -3,57 | 32,5 | 12 | -1,16 | - | 49,0 | 2,3 |
| 11-1 | Before | L | 38+0 | Full term | C section | f | 2890 | Normal BW | 25 | -0,67 | 47 | 7 | -1,48 | 33,0 | 16 | -1,00 | 9 | 10 | 10 | - | - | 3750 | 39 | -0,29 | 52,0 | 36 | -0,37 | 36,5 | 67 | 0,44 | - | 38,0 | 16,9 |
| 12-1 | With | M | 18+3 | | | | | | | | | | | | | | | | | | | | | | | | | | | | | | |
| 12-2 | After | L | 34+5 | Late PT | Vaginal | f | 1940 | Low BW | 19,00 | -0,87 | 44 | 36,00 | -0,35 | 31,0 | 44,00 | -0,16 | 9 | 9 | 10 | - | - | 2482 | 4 | -1,77 | 48,0 | 27 | -0,62 | 33,0 | 23 | -0,74 | - | 28,0 | 3,4 |
| 13-1 | Before | L | 39+0 | Full term | C section | m | 3760 | Normal BW | 74 | 0,64 | 53 | 68 | 0,48 | 33,0 | 4 | -1,77 | 9 | 10 | 10 | - | Yes | 4410 | 75 | 0,69 | 56,0 | 92,0 | 1,4 | 37,5 | 82 | 0,92 | Yes | 26,0 | 18,2 |
| 13-2 | After | L | 38+3 | Full term | C section | m | 3030 | Normal BW | 21 | -0,82 | 51 | 42 | -0,19 | 33,0 | 6 | -1,53 | 9 | 10 | 10 | - | Yes | 4860 | 63 | 0,32 | 56,0 | 63 | 0,34 | na | na | na | - | 45,0 | 7,2 |
| 13-3 | With | L | 38+0 | Full term | C section | f | 3240 | Normal BW | 56 | 0,15 | 49 | 27 | -0,61 | 34,0 | 41 | -0,23 | 9 | 10 | 10 | - | - | 4540 | 81 | 0,89 | 55,0 | 83 | 0,97 | 36,5 | 59 | 0,24 | - | 40,0 | 5,1 |
| 14-1 | Before | M | <12 | | | | | | | | | | | | | | | | | | | | | | | | | | | | | | |
| 14-2 | Before | L | 40+0 | Full term | Vaginal | m | 3950 | Normal BW | 77 | 0,74 | 53 | 50 | 0,00 | na | na | na | na | na | na | - | - | na | na | na | na | na | na | na | na | na | Yes | | 43,9 |
| 14-3 | Before | L | 43+1 | Full term | Vaginal | f | 3550 | Normal BW | 44 | -0,15 | 49 | 7 | -1,47 | 34,0 | 15 | -1,05 | 10 | 10 | na | - | - | 3900 | 9 | -1,33 | 53,0 | 12 | -1.19 | na | na | na | Yes | 29,0 | 42,2 |
| 14-4 | Before | M | <12 | | | | | | | | | | | | | | | | | | | | | | | | | | | | | | |
| 14-5 | Before | L | 41+0 | Full term | Vaginal | f | 3900 | Normal BW | 77 | 0,75 | 50 | 16 | -1,00 | 35,0 | 44 | -0,15 | 9 | 10 | 10 | - | - | 4900 | 72 | 0,57 | na | na | na | na | na | na | Yes | 34,0 | 36,5 |
| 14-6 | Before | L | 38+6 | Full term | Vaginal | m | 3565 | Normal BW | 59 | 0,24 | 51 | 37 | -0,34 | 34,0 | 17 | -0,95 | 9 | 10 | 10 | - | - | 4260 | 49 | -0,03 | 54,0 | 48 | -0,05 | 38,0 | 82 | 0,92 | Yes | 33,0 | 27,2 |
| 15-1 | Before | M | <12 | | | | | | | | | | | | | | | | | | | | | | | | | | | | | | |
| 15-2a | With | L | 31+5 | Very PT | C section | f | 1340 | Very low BW | 22,00 | -0,78 | 38 | 14,00 | -1,08 | 28,0 | 35,00 | -0,38 | 7 | 9 | 9 | Yes | Yes | 2790 | 16 | -1,01 | 49,5 | 58 | 0,19 | 33,9 | 51 | 0,02 | - | 42,0 | 2,5 |
| 15-2b | With | L | 31+5 | Very PT | C section | f | 1495 | Very low BW | 36,00 | -0,35 | 40 | 31,00 | -0,51 | 29,5 | 74,00 | 0,64 | 8 | 9 | 9 | Yes | - | 2920 | 24 | -0,7 | 52,0 | 94 | 1,53 | 34,4 | 67 | 0,44 | - | 42,0 | 2,5 |
| 16-1 | With | L | 40+6 | Full term | C section | f | 4450 | Normal BW | 98 | 2,07 | 53 | 66 | 0,40 | 38,0 | 99 | 2,19 | 8 | 10 | 10 | Yes | - | 6150 | 85 | 1,04 | 63,0 | 99 | 2,42 | 42,0 | 100 | 2,7 | - | 63,0 | 9,1 |
| 16-2 | After | L | 37+3 | Full term | C section | f | 3130 | Normal BW | 56 | 0,16 | 49 | 35 | -0,38 | 35,0 | 75 | 0,68 | 7 | 7 | 9 | - | - | 5350 | 93 | 1,50 | 60,0 | 100 | 2,87 | 39,5 | 99 | 2,2 | Yes | 53,0 | 6,6 |
| 16-3 | After | L | 38+0 | Full term | C section | f | 3150 | Normal BW | 48 | -0,06 | 51 | 60 | 0,26 | 33,5 | 27 | -0,62 | 6 | 8 | 9 | - | - | na | na | na | na | na | na | na | na | na | Yes | na | 5,5 |
| 16-4 | After | L | 37+5 | Full term | C section | f | 3070 | Normal BW | 45 | -0,12 | 53 | 89 | 1,22 | 34,0 | 44 | -0,14 | 8 | 10 | 10 | - | - | 4610 | 63 | 0,33 | 56 | 78 | 0,78 | 39 | 96 | 1,72 | Yes | 52,0 | 0,1 |
| 17-1 | After | L | 37+3 | Full term | Vaginal | m | 3110 | Normal BW | 42 | -0,19 | 51 | 57 | 0,17 | 33,0 | 13 | -1,11 | 9 | 10 | 10 | - | - | 4100 | 66 | 0,40 | 54,5 | 81 | 0,89 | 37,5 | 88 | 1,16 | - | 36,0 | 10,9 |
| 17-2 | After | L | 40+3 | Full term | Vaginal | m | 3956 | Normal BW | 74 | 0,64 | 52 | 37 | -0,33 | 36,0 | 58 | 0,21 | 9 | 10 | 10 | - | - | 5900 | 93 | 1,48 | 55,0 | 29 | -0,56 | 39,0 | 81 | 0,87 | - | 37,0 | 9,1 |
| 17-3 | After | S | 33+3 | Moderate PT | Vaginal | m | 2230 | Low BW | | | 46 | | | 35,0 | | | | | | | | | | | | | | | | | | | |
| 17-4 | After | L | 36+0 | Late PT | Vaginal | m | 3410 | Normal BW | 94,00 | 1,54 | 49 | 77,00 | 0,74 | 36,0 | 99,00 | 2,25 | 7 | 8 | 9 | - | - | 5180 | 99 | 2,38 | 55,0 | 92 | 1,39 | 40,0 | 100 | 3,54 | - | 41,0 | 4,6 |
| 17-5 | After | L | 38+2 | Full term | Vaginal | m | 3950 | Normal BW | 91 | 1,36 | 52 | 61 | 0,29 | 35,0 | 50 | -0,01 | 9 | 10 | 10 | Yes | - | 4970 | 92 | 1,43 | 57,5 | 98 | 2,06 | 38,5 | 95 | 1,63 | - | 33,0 | 1,4 |
| 18-1 | With | L | 39+1 | Full term | Vaginal | m | 2685 | Normal BW | 3 | -1,89 | 50 | 19 | -0,86 | 33,0 | 4 | -1,80 | na | na | 10 | Yes | - | 3930 | 35 | -0,38 | 53,0 | 37 | -0,34 | 38,0 | 87 | 1,13 | - | 31,0 | 2,9 |
| 19-1 | Before | L | 41+0 | Full term | Vaginal | m | 3200 | Normal BW | 12 | -1,19 | na | na | na | na | na | na | na | na | na | - | - | na | na | na | na | na | na | na | na | na | Yes | na | 31,0 |
| 19-2 | Before | L | 40+0 | Full term | Vaginal | m | 3200 | Normal BW | 17 | -0,96 | na | na | na | na | na | na | na | na | na | - | - | na | na | na | na | na | na | na | na | na | Yes | na | 29,5 |
| 20-1 | After | M | <12 | | | | | | | | | | | | | | | | | | | | | | | | | | | | | | |
| 20-2 | After | L | 38+2 | Full term | Vaginal | m | 3170 | Normal BW | 33 | -0,44 | 52 | 61 | 0,29 | 34,0 | 23 | -0,74 | 9 | 10 | 10 | - | - | 4490 | 32 | -0,46 | 57,0 | 75 | 0,66 | 38,0 | 57 | 0,18 | Yes | 49,0 | 0,3 |
| 21-1 | Before | L | 41+3 | Full term | C section | f | 4090 | Normal BW | 87 | 1,12 | 52 | 44 | -0,15 | 35,0 | 41 | -0,22 | 9 | 10 | 10 | - | - | 4900 | 63 | 0,32 | 57,0 | 77 | 0,75 | 37,0 | 34 | -0,41 | - | 35,0 | 17,2 |
| 21-2 | Before | L | 40+2 | Full term | C section | m | 3560 | Normal BW | 41 | -0,22 | 50 | 12 | -1,16 | 36,0 | 59 | 0,24 | 9 | 10 | 10 | - | - | 3950 | 14 | -1,08 | 55,0 | 49 | -0,03 | 37,0 | 35 | -0,38 | Yes | 31,0 | 15,3 |
| 22-1 | After | L | 39+1 | Full term | Vaginal | f | 3010 | Normal BW | 20 | -0,84 | 53 | 79 | 0,82 | 33,0 | 10 | -1,26 | 9 | 10 | 10 | - | - | 4000 | 46 | -0,10 | 56,0 | 92 | 1,42 | 36,0 | 40 | -0,25 | - | 33,0 | 11,3 |
| 22-2 | After | L | 38+2 | Full term | Vaginal | m | 3340 | Normal BW | 48 | -0,05 | 50 | 28 | -0,58 | 33,0 | 7 | -1,47 | 9 | 10 | 10 | - | Yes | 4830 | 64 | 0,35 | 55,0 | 46 | -0,1 | 38,5 | 80 | 0,83 | Yes | 45,0 | 8,2 |
| 22-3 | After | L | 40+3 | Full term | Vaginal | m | 3720 | Normal BW | 54 | 0,11 | 52 | 37 | -0,33 | 35,0 | 29 | -0,56 | 9 | 10 | 10 | - | - | 4850 | 33 | -0,45 | 57,5 | 61 | 0,28 | 39.5 | 82 | 0,93 | Yes | 44,0 | 2,3 |
| 23-1 | Before | L | na | na | C section | m | na | na | na | na | na | na | na | na | na | na | na | na | na | na | na | na | na | na | na | na | na | na | na | na | na | na | 19,6 |
| 23-2 | Before | L | na | na | C section | na | na | na | na | na | na | na | na | na | na | na | na | na | na | na | na | na | na | na | na | na | na | na | na | na | na | na | 15,0 |
| 24-1 | Before | L | 40+3 | Full term | Vaginal | m | na | na | na | na | na | na | na | na | na | na | na | na | na | - | na | na | na | na | na | na | na | na | na | na | na | na | 18,7 |
| 24-2 | With | L | 35 | Late PT | Vaginal | m | na | na | na | na | na | na | na | na | na | na | na | na | na | - | na | na | na | na | na | na | na | na | na | na | na | na | 11,8 |
| 25-1 | After | L | 39+2 | Full term | Vaginal | m | 3130 | Normal BW | 18 | -0,91 | 51 | 32 | -0,47 | 35,0 | 38 | -0,30 | 9 | 10 | 10 | - | Yes | 4910 | 96 | 1,81 | 58 | 100 | 2,8 | 38 | 95 | 1,67 | Yes | 20,0 | 0,4 |
| 26-1 | With | L | 41+2 | Full term | Vaginal | m | 3420 | Normal BW | 23 | -0,74 | 51 | 17 | -0,94 | 35,0 | 24 | -0,72 | 9 | 10 | 10 | - | - | 4215 | 19 | -0,87 | 55,5 | 48 | -0,04 | 39,5 | 94 | 1,52 | - | 29,0 | 0,9 |
| 27-1 | Before | M | na | | | | | | | | | | | | | | | | | | | | | | | | | | | | | | |
| 27-2 | Before | M | na | | | | | | | | | | | | | | | | | | | | | | | | | | | | | | |
| 27-3 | Before | A (i) | 6 | | | | | | | | | | | | | | | | | | | | | | | | | | | | | | |
| 27-4 | Before | L | 38+2 | Full term | C section | m | 3485 | Normal BW | 61 | 0,29 | 49 | 16 | -1,01 | 34,5 | 35 | -0,38 | 9 | 10 | 10 | - | - | 4820 | 95 | 1,67 | 54,3 | 81 | 0,87 | 36,0 | 48 | -0,04 | Yes | 27,0 | 6,9 |
| 27-5 | Before | M | na | | | | | | | | | | | | | | | | | | | | | | | | | | | | | | |
| 27-6 | Before | M | na | | | | | | | | | | | | | | | | | | | | | | | | | | | | | | |
| 27-7 | Before | M | na | | | | | | | | | | | | | | | | | | | | | | | | | | | | | | |
| 27-8 | Before | A (i) | 9+1 | | | | | | | | | | | | | | | | | | | | | | | | | | | | | | |
| 27-9 | With | A (i) | 20 | | | | | | | | | | | | | | | | | | | | | | | | | | | | | | |
| 28-1 | Before | L | 40+0 | Full term | Vaginal | m | 4100 | Normal BW | 86 | 1,08 | 55 | 86 | 1,09 | na | na | na | 9 | 10 | 10 | - | - | na | na | na | na | na | na | na | na | na | Yes | na | 47,7 |
| 28-2 | Before | A (i) | 6 | | | | | | | | | | | | | | | | | | | | | | | | | | | | | | |
| 28-3 | Before | A (i) | 6 | | | | | | | | | | | | | | | | | | | | | | | | | | | | | | |
| Life birth missing data | **0** | **0** | 3 | 3 | 0 | 1 | 5 | 5 | 5 | 5 | 7 | 7 | 7 | 9 | 9 | 9 | 10 | 10 | 12 | 2 | 5 | 11 | 11 | 11 | 12 | 12 | 12 | 14 | 14 | 14 | 7 | 10 | 0 |

Miscarriages, abortions and stillbirths marked grey

cTMA, complement mediated thrombotic microangiopathy
M, miscarriage; A(i), induced abortion; L, live birth; S, stillbirth
PT, preterm; na, not available
m, male; f, female
BW, birth weight
NICU, neonatal intensive care unit

Malformations include: hemangioma, hip dysplasia (2 infants), patent foramen ovale & atrioseptal defect 2, pigmeted mole, pigeon toes, pigeon toes & frenulum of tongue

**Table S5.** Characteristics of 46 live births according to infant sex[a]**.**

| Characteristic | Female infants | Male infants |
|---|---|---|
| Women, n | 14 | 19 |
| Infants, n | 19[b] | 27 |
| Gestational age (weeks) | 38 (35, 41) | 39 (38, 40) |
| Term vs. preterm | | |
| Full term | 12 (63) | 23 (85) |
| Late preterm | 2 (11) | 3 (11) |
| Moderate preterm | 1 (5) | 0 |
| Very preterm | 2 (11) | 0 |
| Extremely preterm | 1 (5) | 0 |
| Mode of delivery, | | |
| Caesarean section | 12 (63)[b] | 8 (30) |
| Vaginal delivery | 7 (37) | 19 (70) |
| Induction of labor | 2 (29) | 7 (37) |
| Weight at birth | | |
| Grams | 3085 (2043, 3435) | 3353 (3120, 3643) |
| Centile | 45 (22, 56) | 45 (18, 68) |
| Birth weight categories | | |
| Normal birth weight | 12 (63) | 23 (85) |
| Low birth weight | 2 (11) | 1 (4) |
| Very low birth weight | 3 (16) | 0 |
| Extremely low birth weight | 1 (5) | 0 |
| Size for gestational age | | |
| Small | 0 | 3 (11) |
| Appropriate | 17 (90) | 19 (70) |
| Large | 1 (5) | 2 (7) |
| Length at birth | | |
| Centimeter | 49 (44, 51) | 51 (50, 52) |
| Centile | 33 (14, 60) | 40 (28, 61) |
| Head circumference at birth | | |
| Centimeter | 34 (31, 34) | 34 (33, 36) |
| Centile | 41 (25, 54) | 32 (10, 56) |
| Apgar score | | |

| | | |
|---|---|---|
| 1 minute | 9 (8, 9) | 9 (9, 9) |
| 5 minutes | 10 (9, 10) | 10 (10, 10) |
| 10 minutes | 10 (9, 10) | 10 (10, 10) |
| Admission to NICU | 5 (26) | 2 (7) |
| Malformations | 2 (11) | 5 (19) |
| Number of neonates per pregnancy condition | | |
| Before cTMA | 7 (37) | 14 (52) |
| With cTMA | 6 (32) | 3 (11) |
| After cTMA | 6 (32) | 10 (37) |
| Breastfeeding | 5 (26) | 15 (56) |

Data are given as n (%) or median (p25, p75)

[a], excluding one with unknown sex and mother lost to follow-up

[b], including one set of twins

n, number; NICU, neonatal intensive care unit; cTMA; complement mediated thrombotic microangiopathy

**Table S6.** Neonatal outcomes of 46 infants born **a)** before manifestation of cTMA (n=22), **b)** related to a cTMA onset or recurrence (n=9, including one set of female twins), **c)** after manifestation of cTMA (n=16) according to neonatal sex [a].

**a)**

| | Female infants (n=7) | | Male infants (n=14) | |
|---|---|---|---|---|
| Characteristic | Birth | Follow-up | Birth | Follow-up |
| Age at follow-up (days) | - | 36 (29-38) | - | 31 (25-67) |
| Weight (g) | 3525 (2890-4090) | 4365 (3750-4900) | 3425 (2117-4100) | 4410 (2880-5150) |
| Weight (centile) | 45 (19-87) | 49 (9-72) | 45 (1-86) | 49 (14-95) |
| Weight gain velocity (g/week) | - | 169 (85-239) | - | 214 (88-346) |
| Length (cm) | 50 (47-52) | 53(52-57) | 52 (46-55) | 54 (50-56) |
| Length (centile) | 12 (7-44) | 36 (12-84) | 44 (11-86) | 53 (<1-92) |
| Length gain velocity (cm/week) | - | 1.0 (0.8-1.4) | - | 0.8 (0.3-1.4) |
| Head circumference (cm) | 34 (33-35) | 37 (37-39) | 34 (33-37) | 38 (35-39) |
| Head circumference (centile) | 22 (15-44) | 63 (34-95) | 44 (1-85) | 66 (10-93) |
| Head growth (cm/week) | - | 0.7 (0.4-0.9) | - | 0.6 (0.2-1.2) |
| NICU | 0 | - | 0 | - |
| Breastfeeding | - | 2 (29) | - | 10 (71) |

**b)**

| | Female infants (n=6) | | Male infants (n=3) | |
|---|---|---|---|---|
| Characteristic | Birth | Follow-up | Birth | Follow-up |
| Age at follow-up (days) | - | 42 (25-63) | - | 30 (29-31) |
| Weight (g) | 1418 (888-4450) | 2855 (900-6150) | 3053 (2685-3420) | 4073 (3930-4215) |
| Weight (centile) | 39 (11-98) | 20 (<1-85) | 13 (3-23) | 27 (19-35) |
| Weight gain velocity (g/week) | - | 208 (3.4-242) | - | 236 (192-281) |
| Length (cm) | 39 (35-53) | 51 (35-63) | 51 (50-51) | 54 (53-56) |
| Length (centile) | 29 (11-66) | 71 (<1-99) | 18 (17-19) | 43 (37-48) |
| Length gain velocity (cm/week) | - | 1.1 (0-2.1) | - | 0.9 (0.7-1.1) |
| Head circumference (cm) | 29 (24-38) | 34 (25-42) | 34 (33-35) | 39 (38-40) |
| Head circumference (centile) | 48 (35-99) | 55 (<1-100) | 14 (4-24) | 91 (87-94) |
| Head growth (cm/week) | - | 0.5 (0.1-1.0) | - | 1.1 (1.1-1.1) |
| NICU | 5 (83) | - | 1 (33) | - |
| Breastfeeding | - | 0 | - | 0 |

**c)**

| | Female infants (n=6) | | Male infants (n=10) | |
|---|---|---|---|---|
| Characteristic | Birth | Follow-up | Birth | Follow-up |
| Age at follow-up (days) | - | 43 (28-53) | - | 43 (20-49) |
| Weight (g) | 3040 (1940-3150) | 4000 (2482-5350) | 3255 (3110-3956) | 4855 (4100-5900) |
| Weight (centile) | 47 (19-56) | 46 (4-93) | 48 (18-94) | 67 (32-99) |
| Weight gain velocity (g/week) | - | 207 (136-293) | - | 224 (180-623) |
| Length (cm) | 50 (44-53) | 56 (48-60) | 51 (49-52) | 56 (55-58) |
| Length (centile) | 70 (35-89) | 78 (27-100) | 45 (28-77) | 78 (29-100) |
| Length gain velocity (cm/week) | - | 0.6 (0.4-1.5) | - | 0.8 (0.6-2.5) |
| Head circumference (cm) | 33 (31-35) | 36 (33-40) | 35 (33-36) | 39 (36-40) |
| Head circumference (centile) | 44 (10-85) | 40 (23-99) | 34 (6-99) | 82 (26-100) |
| Head growth (cm/week) | - | 0.6 (0.2-0.7) | - | 0.7 (0.3-1.1) |
| NICU | 0 | - | 1 (10) | - |
| Breastfeeding | - | 3 (50) | - | 5 (50) |

Data are given as n (%) or median (full range)

[a], excluding one with unknown sex and mother lost to follow-up

NICU, neonatal intensive care unit; cTMA; complement mediated thrombotic microangiopathy
